# The relationship between dietary sulfur amino acids intake and severity and frequency of pain in Iranian patients with musculoskeletal pains, 2020

**DOI:** 10.1186/s13104-021-05899-9

**Published:** 2022-01-10

**Authors:** Niki Bahrampour, Ariyo Movahedi, Abolghassem Djazayery, Cain C. T. Clark

**Affiliations:** 1 Department of Nutrition, Science and Research Branch, Islamic Azad University, Tehran, Iran; 2 Department of Community Nutrition, School of Nutritional Sciences and Dietetics, Tehran University of Medical Sciences (TUMS), Tehran, Iran; 3 Centre for Intelligent Healthcare, Coventry University, Coventry, CV1 5FB UK

**Keywords:** Musculoskeletal pain, Sulfur amino acids, Diet, Pain

## Abstract

**Objective:**

Musculoskeletal pain conditions (MPs) are a widespread public problem that can affect 13.5% to 47% of the total population. Dietary changes can have strong effects on person’s health; for instance, Sulfur amino acids (SAAs) can act as a precursor of neurotransmitters, antioxidative metabolic intermediates, such as glutathione, impact inflammation, and play a role in severity and frequency of MPs. We evaluated the relationship between dietary SAAs intake with severity and frequency of pain in patients with MPs.

**Results:**

This cross-sectional study consisted of 175 men and woman. Anthropometric measurements and pain assessments were conducted via questionnaires. Dietary data were collected using 7 days 24-h recall. ANOVA and Spearman correlation coefficients were used to examine the relationship and correlation, respectively, between exposure and outcome variables.

There was a significant correlation between age, weight, waist circumference (WC), waist circumference to height (WHtR), body mass index (BMI), and severity and frequency of MPs among women. There was a correlation between age and severity of pain in men.

The present study highlights a positive association between the dietary SAAs and severity of pain, even after adjusting for confounding variables.

**Supplementary Information:**

The online version contains supplementary material available at 10.1186/s13104-021-05899-9.

## Introduction

Musculoskeletal pain conditions (MPs) are a widespread public problem, and there is an urgent and on-going need to control and manage severity and frequency of pain in patients. MPs are multifaceted and affect 13.5% to 47% of total population [1, 2]; indeed, MPs impose great financial pressure, both personally and to healthcare systems [3]. Lower back, neck, shoulders, and knee pains are major causes of disability throughout life [4–6]. Empirical evidence suggests that most treatments, although provide some short-term relief, have little long-term efficacy for MPs [6, 7].

Recently, lifestyle changes, unhealthy diet, and obesity have been considered to further managing chronic pain [5]. Indeed, dietary changes have multiple effects (positive or negative) on a person’s health [8]. Sulfur amino acids (SAAs), as a part of protein structure, [9] can act as a precursor of neurotransmitters and antioxidative metabolic intermediates such as glutathione. Moreover, SAA’s can play a role in inflammation, severity and frequency of MPs [10]. Indeed, it is evident the amount of SAAs received from daily diet is inappropriate with individual needs. Accordingly, some previous studies have reported that SAAs consumption may have an alleviating action on a large number of chronic diseases and degenerative changes, which are associated with normal aging, by inhibiting oxidative stress [11]. One of the main role of SAAs is necessary for detoxification of many drugs, such as acetaminophen, which is widely used for relieving pain by the liver [9]. However, to our knowledge, no studies have been performed to suitably investigate the potential association between SAA’s and MP’s. In the present study, we sought to examine the relationship between dietary SAAs intake and severity and frequency of pain in Iranian patients with MPs.

## Main text

### Materials and methods

#### Study population

This study was designed as a cross-sectional study and done from February to October 2020, with multistage cluster random sampling. The target population were volunteers referred to physiotherapy and orthopedic clinics in districts 2 and 3 of Tehran, Iran. Participants who were enrolled in the study consisted of 175 men and woman above 18 years. Having a bone fracture in last 3 months, pregnancy and lactation, and psychosomatic disorders were the exclusion criteria. Age, sex, education status, occupation, smoking status, menopausal status, number of births, and delivery type(s) were assessed through a demographic questionnaire.

#### Anthropometric measuring

Height and weight were measured, using a Seca 216 stadiometer to the nearest 0.1 cm, and Seca scale to the nearest 0.1 kg, respectively, while participants wore light clothes and were unshod. Waist circumference (WC) was measured according to standard procedures by an anthropometrist. Body mass index (BMI) was calculated as weight in kg, divided by height in m^2^.

#### Pain assessment

The validated McGill Pain Questionnaire, consisting of 20 questions, was used to assess the severity of pain information. Scores ranged from 0 (no pain) to 78 (severe pain) [12]. Pain subcategories were pain sensory, pain affective, pain miscellaneous, pain evaluative, and pain frequency. Pain intensity might be expressed by respondent’s questions choice. The frequency of pain was evaluated by the number of days feeling pain per week. All measurements were done by a qualified clinician.

#### Dietary assessment

Dietary data were collected using 7 days 24-h dietary recall through face-to-face interview. Participants were asked to recall all foods and beverages consumed in the preceding 7 days [13]. Portion-sizes of consumed foods were converted to grams, then, each food and beverage was analyzed for their energy and nutrients, especially sulfur-containing amino acids (methionine, cysteine) content, using Nutritionist IV (version 7.0; N-Squared Computing, Salem, OR) Software modified for Iranian foods [14]. The software database was drawn from United States Department of Agriculture (USDA) food composition tables. Total energy intake between 800 and 4000 kcal was accepted, with intake outside of the range resulting in exclusion form the analysis. Participant's total SAAs intake was divided into tertiles based on trend of dietary SAAs.

#### Statistical analysis

Data analysis was performed using the SPSS version 26 (IBM SPSS Inc.). The Kolmogorov–Smirnov test was used to evaluate the normality of the data, and mean and SD (standard deviation) and median (mid-quarter range) were used to describe quantitative variables, whilst frequency report (percentage) was used for qualitative variables. Analysis of variance (ANOVA), Chi-square, and analysis of covariance (ANCOVA) were used to compare qualitative and quantities factors between SAAs tertiles. Spearman correlation coefficients were computed to examine the correlation between independent and outcome variables. Linear logistic regression was used to find the relationship between SAAs intake and pain. We computed 3 models: crude model, model 1 (adjusted for age, PA, energy intake, BMI, WC), and a final model (adjusted for model 1 + gender, education, job, marital status, and delivery type). For data analysis, a P-value < 0.05 was, a priori, considered statistically significant.

### Results

#### Study population and general characteristics

General characteristics of participants are shown across SAAs tertiles in Table 1. The mean ± SD of BMI and daily energy intake were 24.84 ± 4.32 kg/m^2^ and 2230 ± 651.71 kcal/day, respectively. No severe pain was reported.

**Table 1:** Characteristics of study participants among total SAAs intake tertiles

Variables	T1 (n = 58)	T2 (n = 59)	T3 (n = 58)
Total SAAs (mg/day)**			
Median	1981.29	2968.39	4646.96
Age (y)*	18.5 ± 5	20 ± 10	25 ± 15
Gender, n (%)**			
Men	5 (8.5)	17 (29.3)	28 (48.3)
Woman	54 (91.5)	41 (70.7)	30 (51.7)
Smoking, n (%)			
Yes	5 (8.5)	12 (20.7)	12 (15.5)
No	54 (91.5)	40 (69.0)	47 (81.0)
Before	0	6 (10.3)	2 (3.4)
Education, n (%)			
Diploma or less	9 (15.3)	6 (10.3)	9 (15.5)
Bachelor degree	32 (54.2)	20 (34.5)	22 (37.9)
Master degree	9 (15.3)	20 (34.5)	16 (27.6)
Phd or higher	9 (15.3)	12 (20.7)	11 (19.0)
BMI (kg/m^2^)	24.51 ± 4.03	25.56 ± 4.65	24.46 ± 4.27
Weight (kg)*	64.50 ± 11.47^a^	72.71 ± 17.21	70.93 ± 15.38
Height (cm)**	162.18 ± 5.63^a^	168.00 ± 9.44	169.86 ± 9.10
Waist circumference (cm)	83.93 ± 15.76	88.79 ± 20.12	87.22 ± 19.96
WHtR			
Normal	59.3	62.1	56.9
At risk	40.7	37.9	43.1
Total pain result	13.51 ± 16.97	14.64 ± 15.59	12.98 ± 14.38
Pain subcategories			
Pain sensory	7.88 ± 9.94	8.17 ± 9.22	7.90 ± 8.79
Pain affective	1.71 ± 2.46	2.31 ± 2.79	1.62 ± 2.29
Pain miscellaneous	2.88 ± 3.96	2.88 ± 3.34	2.26 ± 3.03
Pain evaluative	1.03 ± 1.47	1.28 ± 1.65	1.21 ± 1.55
Pain frequency	1.90 ± 2.38	1.79 ± 1.98	1.53 ± 1.74
Occupation, n (%)			
Manager	5 (8.5)	7 (12.1)	11 (19.0)
Employee	17 (28.8)	12 (20.7)	10 (17.2)
Worker	1 (1.7)	0	1 (1.7)
Housewife	9 (15.3)	7 (12.1)	0.0
Pensionary	5 (8.5)	4 (6.9)	4 (6.9)
Other	9 (15.3)	8 (13.8)	15 (25.9)
Collegian	10 (16.9)	16 (27.6)	17 (29.3)
No work	3 (5.1)	4 (6.9)	0
Delivery number, n (%)			
0	35 (59.3)	44 (75.9)	50 (86.2)
1	5 (8.5)	5 (8.6)	2 (3.4)
2	11 (18.6)	7 (12.1)	4 (6.9)
3	8 (13.6)	0	1 (1.7)
4	0	1 (1.7)	1 (1.7)
9	0	1 (1.7)	0
Delivery type, n (%)			
Natural childbirth	6 (10.2)	2 (3.4)	4 (6.9)
Cesarean section	18 (30.5)	12 (20.7)	4 (6.9)
Men	5 (8.5)	16 (27.6)	26 (44.8)
No delivery	30 (50.8)	28 (48.3)	24 (41.4)
Menopause, n (%)**	9 (15.3)	4 (6.9)	4 (6.9)
Acetaminophen (325/daily), n (%)	4 (0.2)	7 (0.8)	5 (0.5)

*SD* Standard deviation, *BMI* body mass index, *WHtR* waist to height ratio, *SAAs* sulfur amino acids

Quantitative variables were showed by means ± SD and qualitative variables were showed by number (percentage)

^a^ indicates differences between tertiles

^*^P value < 0.05 and **P value < 0.001 were considered.

P values resulted from ANCOVA analysis and Chi-square test and were adjusted for energy intake

#### Comparison of daily nutrient intake in participants across people in pain tertiles

In Table 2, the intakes of food groups and nutrients are shown after adjusting for energy intake among pain tertiles. The first tertile group includes healthy people with no pain. No difference was found between dietary methionine and cysteine among the three groups (P > 0.05).

**Table 2:** Comparison of daily nutrient intake in participants across people in pain tertiles

Variables Amounts per day	T1 (n = 58)	T2 (n = 59)	T3 (n-58)
Energy (kcal)*	2147.64 ± 670.41	2388.54 ± 629.30	2191.66 ± 601.17
Protein (g)	93.76 ± 57.44	106.85 ± 87.66	88.29 ± 31.64
Carbohydrate (g)*	229.45 ± 94.32	266.99 ± 108.42	253.79 ± 102.37
Fat (g)	102.40 ± 44.93	108.10 ± 42.31	100.52 ± 24.23
Cholesterol (mg)	345.89 ± 253.87	350.19 ± 280.76	337.19 ± 269.04
SFA (g)	29.99 ± 23.46	27.27 ± 9.56	29.07 ± 14.23
PUFA (g)	25.90 ± 13.82	30.71 ± 23.26	25.89 ± 7.28
alpha-Linolenic acid (mg)	3.21 ± 8.98	3.30 ± 9.35	4.45 ± 9.47
Sodium (mg)*	1790.44 ± 831.05	2127.07 ± 1194.83	1859.41 ± 825.34
Potassium (mg)	3670.49 ± 1485.21	3912.67 ± 1530.67	3966.11 ± 1702.20
Calcium (mg)	1487.34 ± 604.26	1603.42 ± 603.94	1601.29 ± 800.16
Magnesium (mg)	826.86 ± 596.75	812.81 ± 490.32	858.05 ± 562.11
Phosphorus (mg)	1530.49 ± 680.62	1637.88 ± 742.92	1518.96 ± 554.84
Vitamin A (RAE)	2143.24 ± 1378.57	2194.53 ± 1583.94	2187.81 ± 1129.64
B1 (mg)*	1.61 ± 0.64	1.87 ± 0.80	1.70 ± 0.58
B2 (mg)	2.49 ± 1.03	2.65 ± 0.96	2.41 ± 0.89
B3 (mg)	22.48 ± 15.39	26.16 ± 21.88	21.50 ± 7.56
B6 (mg)	2.15 ± 1.11	2.44 ± 1.62	2.31 ± 1.39
B9 (μg)	341.64 ± 179.25	343.23 ± 142.97	405.87 ± 316.22
B12 (μg)	7.01 ± 6.56	6.49 ± 5.27	5.82 ± 4.94
Vitamin D (μg)	2.69 ± 2.04	2.66 ± 2.30	2.34 ± 1.99
Fiber (g)	13.99 ± 9.62	15.53 ± 9.03	17.75 ± 15.59
Fructose (g)	7.18 ± 6.12	7.88 ± 5.51	8.79 ± 9.80
Sucrose (g)	15.44 ± 10.88	15.49 ± 8.06	16.47 ± 11.99
Lactose (g)	19.05 ± 12.37	18.61 ± 12.01	15.64 ± 11.11
Tryptophan (mg)	1023.78 ± 654.18	1171.85 ± 1010.08	970.15 ± 347.16
Caffeine (mg)	185.28 ± 132.90	175.90 ± 123.48	226.54 ± 134.59
Methionine (mg)	2164.71 ± 1557.72	2269.54 ± 1479.06	1920.50 ± 773.55
Cysteine (mg)	1176.10 ± 717.85	1285.38 ± 748.37	1128.08 ± 417.79

*SD* standard deviation, *SFA* Saturated fat, *PUFA* Polyunsaturated fatty acids, *SAAs* sulfur amino acids

Data are presented as Mean ± SD

P values resulted from ANCOVA analysis after adjusting energy intake except energy variable

*P-value < 0.05 was significant

#### Relationship between pain frequency and intensity and dietary SAAs

To investigate the correlation between the amount of SAAs and pain, spearman correlation coefficients were computed (Additional file 1: Table S1 and S2). There was a weak correlation between age, weight, WC, WHtR, and BMI and severity and frequency of MPs among women. Similarly, in men, there was an association between age and severity of pain (R = 0.36; P = 0.01). Finally, no relationship was found between SAAs intake tertiles and intensity and frequency of pain among patients with MPs in the crude model and model 1 (shown in Table 3). However, a positive relationship was found, after adjusting all confounders, between pain intensity and SAA intake (β = 3.44, 95% CI 0.05–6.83, P = 0.04).

**Table 3:** Relationship between SAAs intake and intensity and frequency of pain among patients

	SAA intake		
	β	95% CI	P-value
Pain intensity			
Crude	1.21	(− 1.96–4.38)	0.45
*M1*	0.75	(− 3.43–4.94)	0.72
*M2*	3.44	(0.05–6.83)	**0.04**
Pain frequency			
Crude	− 0.18	(− 0.55–0.19)	0.33
*M1*	− 0.16	(− 0.63–0.29)	0.47
*M2*	0.15	(− 0.21–0.53)	0.40

*SAA* sulfur amino acids

M1: adjusted for age, PA, energy intake, BMI, WC

M2: adjusted for M1 + gender, education, job, marital status, delivery type

Logistic regression was used; β regression coefficients refer to SAA intake group's difference

All values are presented as 95% Confidence intervals (95% CI)

P-value < 0.05 was significant

### Discussion

According to the results of the present study, a significant relationship was apparent between SAAs consumption and severity of MPs after adjusting for all confounders. However, there was no relationship between SAAs intake and pain frequency. In addition, 56% of women felt pain more than twice a week. Roughly 60% of the participants were regarded as young, and age was significantly different across among SAAs tertiles [15]. Indeed, aging can affect the progressive loss of neuromuscular function and increase in muscular degenerative injuries [16]. Women reportedly feel pain to a greater extent than men, and much attention has been paid on the effect of sex hormones on pain induction. Microglia plays differently in inducing pain in men and women [17], whilst estrogen receptors influence in synthesis and secretion of the methionine-enkephalin (delta-epithelial ligand receptor) and beta-endorphin secretion [18].

In contrast with this study, Elma et al. found that although the total protein intake was higher than the allowable intake in most patients with pain but the severity of pain was not significantly related to protein intake [8]. There was mostly high SAAs intake among participants, greater than the recommended dietary allowance (14 mg/kg/day) [19]. Most sulfur-rich sources of amino acids are found in animal proteins, which have positive dietary acid load; indeed, according to many studies, this type of protein can lead to metabolic acidosis and inflammation and this may accounted for MPs in this study [20]. An imbalance between acidic and alkaline precursors has been shown to disrupt a chronic net dietary acid load, which may have adverse consequences on bone health and pain [21]. Furthermore, Calder et al. found that excessive consumption of SAAs was associated with increased bone resorption [22], whilst dietary methionine may decrease blood pH and increases skeletal pain [23]. In contrast with this study, no SAAs deficiency was observed in people with arthritis in the study of Freyberg et al., which was conducted to discern the relationship between dietary SAAs and rheumatoid arthritis, [24]. In a cohort study with 546 people with rheumatoid arthritis, no significant correlation was observed between the type of protein consumed and the risk of rheumatoid arthritis [25, 26]. Furthermore, in our study, there was a positive relationship between carbohydrate intake and the group that feel most pain, which may due to induced oxidative stress [27]. In addition, the internal pain relief system in central nervous system requires essential fatty acids, especially Eicosapentaenoic acid (EPA) and Docosahexaenoic acid (DHA) [28]. Whereas the consumption of omega-3 fats (high in fatty fish) was very low in this population. In addition, consumption of sodium was positively correlated with bone pain [29]. One of the possible explanation for such findings is high dietary methionine, which may increase level of homocysteine and could be harmful for musculoskeletal health due to altering nitric oxide (NO) signaling, and endoplasmic reticulum stress [30]. Due to the fact that the consumption of SAAs was high in this study, norepinephrine may have increased chronic pain by producing more inflammatory cytokines [31]. On other hand, SAAs are precursors of S-adenosyl methionine (SAM), hydrogen sulfide, taurine, and glutathione which are protective [32, 33]. Among painkillers drugs, acetaminophen intake was not statistically different among SAAa intake tertiles. Importantly, glutathione detoxifies drugs such as acetaminophen via the sulfur element [34, 35].

In the present study, there was a strong correlation between weight, WC, WHtR, and BMI and the severity and frequency of musculoskeletal pain in women. In addition, the percentage of body fat mass was higher in women, which can be a factor in shortening the height between discs and increasing the severity of pain [36]. In general, excessive consumption of cysteine causes obesity and undesirable metabolic phenotype in mice [37]. Indeed, Stolt et al. noted an increase in serum level of fibroblast growth factor 21 (FGF-21) and lipogenic mRNAs in adipose tissue after a low SAAs diet, which may be further related with reductions in weight, adiposity, and obesity-related diseases [38].

Overall, to our knowledge, this is the first study to have evaluated the relationship between dietary SAAs intake with severity and frequency of pain in patients with MPs in Iran. Indeed, given the volume of SAA’s related supplements sold worldwide, and their potential use in pain management, this study provides a useful basis for further investigation; particularly highlighting the need for elucidation of the safety/risk ratio of SAAs for pain [22].

### Conclusion

In conclusion, the present study shows a positive and relative association between the dietary SAAs and severity of pain. Although, targeting a decrease in dietary animal protein intake (low SAAs diet) and plant-based diet might be a concomitantly useful strategy for reducing pain.

## Limitations

The population of this study was mostly among young adults (18–35 years) and women. Assessing elderly people in quarantine period of covid-19 was logistically difficult and this may influence the final results. In addition, like any cross-sectional study, no causal relationship between exposure and outcome can be discerned. Moreover, some unconsidered confounders may have had a possible influence on our findings. Plant and animal based proteins were not assessed in this study. Indeed, further research, that includes a wide range of ages, should be conducted to clarify our findings.

## Supplementary Information

**Additional file 1:** **Table S1.** Correlation between pain frequency and the studied variables by divided gender. **Table S2.** Correlation between pain intensity and SAAs intake by divided gender.

## Data Availability

However, data are available from the writers with the permission of the clinics and upon fair requests. It has been stated in our contract between the clinic and us that they never send us details about the participants because our data are part of a larger database.

## Abbreviations

SAAs: Sulfur amino acids
RDA: Recommended dietary allowances
WC: Waist circumference
BMI: Body mass index
WHtR: Waist to height ratio
FGF-21: Fibroblast growth factor 21

## References

[1] Gerdle B, Ghafouri B, Ernberg M, Larsson B. Chronic musculoskeletal pain: review of mechanisms and biochemical biomarkers as assessed by the microdialysis technique. J Pain Res. 2014;7:313–26. https://www.pmc/articles/PMC4062547/?report=abstract. Accessed 4 Oct 2020. 10.2147/JPR.S59144 PMC4062547 24966693

[2] Cimmino MA, Ferrone C, Cutolo M (2011). Epidemiology of chronic musculoskeletal pain. Best Prac Res Clinic Rheumatol.

[3] Malik KM, Beckerly R, Imani F. Musculoskeletal disorders a universal source of pain and disability misunderstood and mismanaged: a critical analysis based on the U.S. model of care. Anesthesiol Pain Med. (Vol. 8) Kowsar: Medical Publishing Company. 2018. https://www.pmc/articles/PMC6348332/?report=abstract. Accessed 8 Oct 2020. 10.5812/aapm.85532 PMC6348332 30775292

[4] Shane Anderson A, Loeser RF (2010). Why is osteoarthritis an age-related disease?. Best Pract Res Clin Rheumatol.

[5] Atella V, Piano Mortari A, Kopinska J, Belotti F, Lapi F, Cricelli C (2019). Trends in age-related disease burden and healthcare utilization. Aging Cell.

[6] Babatunde OO, Jordan JL, Van Der Windt DA, Hill JC, Foster NE, Protheroe J (2017). Effective treatment options for musculoskeletal pain in primary care: a systematic overview of current evidence. PLoS ONE.

[7] Chronic Pain - StatPearls - NCBI Bookshelf. 2020. https://www.ncbi.nlm.nih.gov/books/NBK553030/. Accessed 8 Oct 2020.

[8] Elma Ö, Yilmaz ST, Deliens T, Coppieters I, Clarys P, Nijs J, et al. Do nutritional factors interact with chronic musculoskeletal pain? A systematic review. J Clin Med. 2020;9(3):702. https://www.pmc/articles/PMC7141322/?report=abstract. Accessed 21 Nov 2020. 10.3390/jcm9030702 PMC7141322 32150934

[9] Nimni ME, Han B, Cordoba F (2007). Are we getting enough sulfur in our diet?. Nutr Metab.

[10] Loeser RF. The role of aging in the development of osteoarthritis. Trans Am Clinic Climatol Assoc. 2017;128:44–54. http://www.ncbi.nlm.nih.gov/pubmed/28790486. Accessed 29 Sept 2019. PMC5525396 28790486

[11] Ying J, Zhang M, Qiu X, Lu Y (2018). The potential of herb medicines in the treatment of esophageal cancer. Biomed Pharmacother.

[12] Khosravi M, Sadighi S, Moradi S, Zendehdel K. Persian-McGill pain questionnaire; translation, adaptation and reliability in cancer patients: a brief report. Tehran Univ Med J. 2013;71(1):53–8. http://tumj.tums.ac.ir. Accessed 26 Sept 2019.

[13] Akbarzade Z, Mohammadpour S, Djafarian K, Clark CCT, Ghorbaninejad P, Mohtashami M (2020). Breakfast-based dietary patterns and obesity in tehranian adults. J Obes Metab Syndr.

[14] Mouratidou T, Ford FA, Fraser RB (2011). Reproducibility and validity of a food frequency questionnaire in assessing dietary intakes of low-income Caucasian postpartum women living in Sheffield, United Kingdom. Matern Child Nutr.

[15] Lautenbacher S, Peters JH, Heesen M, Scheel J, Kunz M (2017). Age changes in pain perception: a systematic-review and meta-analysis of age effects on pain and tolerance thresholds. Neurosci Biobehav Rev.

[16] Minetto MA, Giannini A, McConnell R, Busso C, Torre G, Massazza G. Common musculoskeletal disorders in the elderly: the star triad. J Clin Med. 2020;9(4). http://www.ncbi.nlm.nih.gov/pubmed/32340331; 10.3390/jcm9041216. 10.3390/jcm9041216 PMC7231138 32340331

[17] Chen G, Zhang Y-Q, Qadri YJ, Serhan CN, Ji R-R. Microglia in pain: detrimental and protective roles in pathogenesis and resolution of pain. Neuron. 2018;100(6):1292–311. https://linkinghub.elsevier.com/retrieve/pii/S0896627318310006; 10.1016/j.neuron.2018.11.009. 10.1016/j.neuron.2018.11.009 PMC6312407 30571942

[18] Aloisi AM. Why we still need to speak about sex differences and sex hormones in pain. Pain Ther. 2017;6(2):111–4. https://www.pmc/articles/PMC5693815/?report=abstract. Accessed 17 Nov 2020. 10.1007/s40122-017-0084-3 PMC5693815 29058253

[19] Nimni ME, Han B, Cordoba F. Are we getting enough sulfur in our diet?. Nutr Metabol. 2007;4:24. https://www.pmc/articles/PMC2198910/?report=abstract. Accessed 6 Dec 2020 10.1186/1743-7075-4-24 PMC2198910 17986345

[20] Hruby A, Jacques PF (2019). Dietary protein and changes in biomarkers of inflammation and oxidative stress in the Framingham Heart Study offspring cohort. Curr Dev Nutr.

[21] Delimaris I. Adverse effects associated with protein intake above the recommended dietary allowance for adults. ISRN Nutr. 2013;2013:1–6. https://www.pmc/articles/PMC4045293/?report=abstract. Accessed 28 Nov 2020. 10.5402/2013/126929 PMC4045293 24967251

[22] Pujos-Guillot E, Pickering G, Lyan B, Ducheix G, Brandolini-Bunlon M, Glomot F, et al. Therapeutic paracetamol treatment in older persons induces dietary and metabolic modifications related to sulfur amino acids. Age (Omaha). 2012;34(1):181–93. https://www.pmc/articles/PMC3260351/?report=abstract. Accessed 28 Nov 2020. 10.1007/s11357-011-9218-4 PMC3260351 21340541

[23] Calder PC, Ahluwalia N, Albers R, Bosco N, Bourdet-Sicard R, Haller D (2013). A consideration of biomarkers to be used for evaluation of inflammation in human nutritional studies. Br J Nutr..

[24] Freyberg RH, Block WD, Fromer MF. A study of sulfur metabolism and the effect of sulfur administration in chronic arthritis 1. J Clin Invest. 1940;19(2):423–35. https://www.ncbi.nlm.nih.gov/pmc/articles/PMC434976/. Accessed 27 Nov 2020. 10.1172/JCI101144 PMC434976 16694758

[25] Benito-Garcia E, Feskanich D, Hu FB, Mandl LA, Karlson EW. Protein, iron, and meat consumption and risk for rheumatoid arthritis: a prospective cohort study. Arthritis Res Ther. 2007;9(1). https://pubmed.ncbi.nlm.nih.gov/17288585/. Accessed 28 Nov 2020. 10.1186/ar2123 PMC1860075 17288585

[26] Hackney KJ, English KL. Protein and essential amino acids to protect musculoskeletal health during spaceflight: evidence of a paradox?. Life. 2014;4:295–317. https://www.pmc/articles/PMC4206848/?report=abstract. Accessed 21 Nov 2020. 10.3390/life4030295 PMC4206848 25370374

[27] Strath LJ, Jones CD, Philip George A, Lukens SL, Morrison SA, Soleymani T, et al. The effect of low-carbohydrate and low-fat diets on pain in individuals with knee osteoarthritis. Pain Med. 2020;21(1):150–60. https://academic.oup.com/painmedicine/article/21/1/150/5380130; 10.1093/pm/pnz022. 10.1093/pm/pnz022 PMC7999621 30865775

[28] Ochi E, Tsuchiya Y. Eicosahexanoic acid (EPA) and docosahexanoic acid (DHA) in muscle damage and function. Nutrients. 2018;10. https://www.pmc/articles/PMC5986432/?report=abstract. Accessed 6 Dec 2020. 10.3390/nu10050552 PMC5986432 29710835

[29] Elma Ö, Yilmaz ST, Deliens T, Coppieters I, Clarys P, Nijs J, et al. Do nutritional factors interact with chronic musculoskeletal pain? A systematic review. J Clin Med. 2020;9(3). http://www.ncbi.nlm.nih.gov/pubmed/32150934; 10.3390/jcm9030702. 10.3390/jcm9030702 PMC7141322 32150934

[30] Stone LS, Molliver DC. In search of analgesia: emerging roles of GPCRs in pain. Mol Interv. 2009;9(5):234–51. http://www.ncbi.nlm.nih.gov/pubmed/19828831; 10.1124/mi.9.5.7. 10.1124/mi.9.5.7 PMC2861805 19828831

[31] Hannibal KE, Bishop MD. Chronic stress, cortisol dysfunction, and pain: a psychoneuroendocrine rationale for stress management in pain rehabilitation. Phys Ther. 2014;94(12):1816–25. https://www.pmc/articles/PMC4263906/?report=abstract. Accessed 6 Dec 2020. 10.2522/ptj.20130597 PMC4263906 25035267

[32] Bin P, Huang R, Zhou X. Oxidation resistance of the sulfur amino acids: Methionine and cysteine. Vol. 2017, BioMed Research International. Hindawi Limited. 2017. 10.1155/2017/9584932 PMC5763110 29445748

[33] Demirkol O, Ercal N. Glutathione. In: Handbook of analysis of active compounds in functional foods. CRC Press; 2012; 68–85. PMID: 26770075. https://www.ncbi.nlm.nih.gov/pmc/articles/PMC4684116/. Accessed 27 Nov 2020.

[34] Ghodoosi N, Mirzababaei A, Rashidbeygi E, Badrooj N, Sajjadi SF, Setayesh L, et al. Associations of dietary inflammatory index, serum levels of MCP-1 and body composition in Iranian overweight and obese women: a cross-sectional study. BMC Res Notes. 2020. https://www.pmc/articles/PMC7684955/?report=abstract. Accessed 27 Dec 2020. 10.1186/s13104-020-05390-x PMC7684955 33228788

[35] Nalamachu S. An overview of pain management: the clinical efficacy and value of treatment. Am J Manag Care. 2013;19(14 Suppl):s261–6. PMID: 24494608. http://www.ncbi.nlm.nih.gov/pubmed/24494608 24494608

[36] Wang Y-XJ, Griffith JF, Zeng X-J, Deng M, Kwok AWL, Leung JCS, et al. Prevalence and sex difference of lumbar disc space narrowing in elderly chinese men and women: osteoporotic fractures in men (Hong Kong) and osteoporotic fractures in women (Hong Kong) studies. Arthritis Rheum. 2013;65(4):1004–10. http://www.ncbi.nlm.nih.gov/pubmed/23335175; 10.1002/art.37857. 10.1002/art.37857 PMC3618501 23335175

[37] Elshorbagy AK, Church C, Valdivia-Garcia M, Smith AD, Refsum H, Cox R. Dietary cystine level affects metabolic rate and glycaemic control in adult mice. J Nutr Biochem. 2012;23(4):332–40. https://www.pmc/articles/PMC3315011/?report=abstract. Accessed 6 Dec 2020. 10.1016/j.jnutbio.2010.12.009 PMC3315011 21543215

[38] Stolt E, Olsen T, Elshorbagy A, Kožich V, van Greevenbroek M, Øvrebø B (2021). Sulfur amino acid restriction, energy metabolism and obesity: a study protocol of an 8-week randomized controlled dietary intervention with whole foods and amino acid supplements. J Transl Med.

