# Supplementary material for: The relationship between dietary sulfur amino acids intake and severity and frequency of pain in Iranian patients with musculoskeletal pains, 2020
Source: BMC Res Notes. 2022 Jan 10;15:13. doi: 10.1186/s13104-021-05899-9 (PMC8744055; doi:10.1186/s13104-021-05899-9)
Supplement: Supplementary file 1 — Additional file 1: Table S1. Correlation between pain frequency and the studied variables by divided gender. Table S2. Correlation between pain intensity and SAAs intake by divided gender. [file 13104_2021_5899_MOESM1_ESM.docx]

**Supplementary information**

**Additional file 1: Table S and 2.**

Correlation between pain frequency and intensity and the studied variables by divided gender is shown in table s and s2.

| **Table S1 Correlation between pain frequency and the studied variables by divided gender** | | | | | | |
| --- | --- | --- | --- | --- | --- | --- |
| **Variables ^a^** | **men** | | **women** | | **total** | |
|  | **R** | **P** | **R** | **P** | **R** | **P** |
| **SAAs intake** | 0.12 | 0.40 | 0.02 | 0.75 | 0.00 | 0.91 |
| **Weight** | 0.08 | 0.58 | 0.22^*^ | **0.01** | 0.08 | 0.29 |
| **Height** | 0.08 | 0.58 | 0.00 | 0.98 | -0.08 | 0.24 |
| **WC** | 0.13 | 0.34 | 0.24^**^ | **0.005** | 0.14 | 0.06 |
| **WHtR** | 0.16 | 0.25 | 0.24^**^ | **0.006** | 0.19^**^ | **0.009** |
| **BMI** | 0.07 | 0.60 | 0.24^**^ | **0.006** | 0.15^*^ | **0.04** |
| **Age** | 0.22 | 0.10 | 0.22^*^ | **0.01** | 0.24^**^ | **0.001** |
| Abbreviations: SAAs: sulfur amino acids, WHtR: waist to height, BMI: body mass index, WC: waist circumference  * Significant relationship less than 0.05  ** Significant relationship less than 0.01  **^a^** variables were adjusted for energy intake.  Analyzes were performed based on Spearman correlation test. | | | | | | |

| **Table S2 Correlation between pain intensity and SAAs intake by divided gender** | | | | | | |
| --- | --- | --- | --- | --- | --- | --- |
| **variable ^a^** | **men** | | **woman** | | **total** | |
|  | **R** | **P** | **R** | **P** | **R** | **P** |
| **Total SAAs intake** | 0.11 | 0.43 | -0.005 | 0.95 | -0.01 | 0.84 |
| **Weight** | 0.14 | 0.30 | 0.28^**^ | **0.001** | 0.12 | 0.10 |
| **Height** | 0.04 | 0.75 | 0.01 | 0.88 | -0.09 | 0.22 |
| **WC** | 0.003 | 0.98 | 0.25^**^ | **0.004** | 0.16^*^ | **0.03** |
| **WHtR** | 0.0001 | 0.99 | 0.24^**^ | **0.006** | 0.18^*^ | **0.01** |
| **BMI** | 0.15 | 0.28 | 0.30^**^ | **0.001** | 0.20^**^ | **0.006** |
| **Age** | 0.36^*^ | **0.01** | 0.36^**^ | **< 0.001** | 0.36^**^ | **< 0.001** |
| Abbreviations: SAAs: sulfur amino acids, WHtR: waist to height, BMI: body mass index, WC: waist circumference  * Significant relationship less than 0.05  ** Significant relationship less than 0.01  **^a^** variables were adjusted for energy intake.  Analyzes were performed based on Spearman correlation test. | | | | | | |
